# Cardiovascular diseases morbidity and mortality among children, adolescents and young adults with dialysis therapy

**DOI:** 10.3389/fpubh.2023.1142414

**Published:** 2023-04-12

**Authors:** Lung-Chih Li, You-Lin Tain, Hsiao-Ching Kuo, Chien-Ning Hsu

**Affiliations:** ^1^Division of Nephrology, Department of Internal Medicine, Kaohsiung Chang Gung Memorial Hospital, Chang Gung University College of Medicine, Kaohsiung, Taiwan; ^2^Institute for Translational Research in Biomedicine, Kaohsiung Chang Gung Memorial Hospital, Chang Gung University College of Medicine, Kaohsiung, Taiwan; ^3^Division of Pediatric Nephrology, Kaohsiung Chang Gung Memorial Hospital, Chang Gung University College of Medicine, Kaohsiung, Taiwan; ^4^Department of Pharmacy, Kaohsiung Chang Gung Memorial Hospital, Kaohsiung, Taiwan; ^5^School of Pharmacy, Kaohsiung Medical University, Kaohsiung, Taiwan

**Keywords:** cardiovascular disease, end-stage kidney disease, dialysis, children, adolescents, young adults

## Abstract

**Background:**

The age-specific burden of cardiovascular disease (CVD) and mortality in pediatric and young adult patients with end-stage kidney disease (ESKD) remains unclear. We aimed to examine the prevalence and incidence of CVD and all-cause mortality in children and adolescents compared with adults with dialysis in Taiwan.

**Methods:**

This retrospective observational cohort study comprised 3,910 patients with more than 2 time point receipts of dialysis therapy in a year, including 156 aged <12 years (children), 250 aged 13–20 years (adolescents), 1,036 aged 21–30 years (young adults) and 2,468 aged 31–40 years (adults) in a large healthcare delivery system in Taiwan (2003–2017). Age groups were classified by the date of first receipt of dialysis therapy. The outcomes include the composite of CVD events and any cause of death. Death-censored Cox proportional hazard models were used to evaluate the composite outcome risk of CVD in the four age groups.

**Results:**

Among patients receiving dialysis treatment, the risk of composite CVD events [HR, 1.63 (1.22–2.19)] and mortality [HR, 1.76 (1.38–2.25)] was greater in children than the dialysis initiated in older patients. Non-atherosclerotic CVD was more prevalent, especially in younger patients, within the first 6 months after the initiation of dialysis. After 6 months of initial dialysis, the risk of atherosclerotic CVD was higher in adults than those for adolescents and children. The magnitude of CVD risk in adolescents who initiated dialysis therapy was higher in females [HR, 2.08 (1.50–2.88)] than in males [HR, 0.75 (0.52–1.10)].

**Conclusion:**

Younger patients undergoing chronic dialysis with a higher risk of CVD events than older patients are associated with a faster onset of non-atherosclerotic CVD and a higher risk of both CVD- and non-CVD-related mortality.

## Introduction

The increasing size of the population of patients with chronic kidney disease (CKD) and CKD-related morbidity and mortality is a great burden to health systems worldwide. Cardiovascular disease (CVD) is a leading cause of death among patients with end-stage kidney disease (ESKD) (1); however, the risk is compounded by additional factors. In adults aged ≥45 years, 87% have CVD reported at the time of ESKD onset, and approximately 50% of deaths are attributed to CVD in the 2016 United States Renal Data System (USRDS). In pediatric patients with ESKD, mortality is attributed to CVD in 23% of children in the United States and up to 50% in other countries (2, 3). However, the spectrum of CVD in children and young adults (aged <40 years) with ESKD remains unclear in Taiwan and worldwide.

Many CVD risk factors, including traditional risk factors (age, lifestyle, left ventricular hypertrophy, dyslipidemia, hypertension, and diabetes mellitus) and novel risk factors for CVD such as inflammation, endothelial dysfunction, sympathetic overactivation, oxidative stress, vascular calcification, and volume overload, are prevalent in patients with CKD (4, 5). In fact, these cardiovascular risks can increase very early in the progression of CKD at an estimated glomerular filtration rate of approximately 75 ml/min and increase continuously with declining kidney function (6). Patients undergoing chronic hemodialysis have multiple comorbidities and many metabolic disorders, causing cardiovascular comorbidity, mortality, including early mortality.

Studies have provided global estimates of the effects of multiple modifiable risk factors of CVD and mortality. Although data linking risk factors with CVD and mortality have mostly been derived from adult patients older than 40 years in the Annual Report on Kidney Disease in Taiwan (7), the impact of modifiable and non-modifiable risk factors on CVD and mortality may vary by age group. The current study aimed to examine and compare the prevalence and incidence of CVD and all-cause mortality among patients starting dialysis for ESKD of different age groups, including children, adolescents, young adults, and adults. Additionally, the risk profile differences in these groups for all-cause mortality and CVD incidence were compared.

## Methods

### Study design

The cohort study was identified based on all patients aged 0–40 years who received dialysis for ESKD between January 1, 2003, and December 31, 2017. Based on the age at the first receipt of dialysis for ESKD, the study cohort was categorized into children (aged <12 years), adolescents (13–20 years), young adults (21–30 years), and adults (31–40 years). Ascertainment of dialysis for ESKD was based on the billing codes for dialysis (Supplementary Table 1). Only patients who had received at least two dialysis modules in a year were included in the study cohort. The index date was defined as the earliest date of receiving dialysis. To identify the incidence of CVD, patients diagnosed with congenital heart disease were excluded (Supplementary Figure 1).

### Data sources

Electronic health record data for this dialysis cohort were obtained from the largest healthcare delivery system in Taiwan, the Chang Gung Memorial Hospital network. This network, with a total of 9,584 beds, delivered approximately 11% of the health services reimbursed by Taiwan's National Health Insurance program in 2018, including over 9.1 million emergency and outpatient department visits and 300,000 hospital admissions (7, 8). These contain data regarding patient diagnoses, demographic characteristics, healthcare procedures, laboratory results, prescriptions and dispensing (9). This study was approved by the institutional review board of the Chang Gung Medical Foundation in Taipei, Taiwan (permit number, 202001176B0).

### Outcomes

CVD was defined using the International Classification of Diseases, Ninth or Tenth Revision (ICD 9/10) for hospitalization discharge codes as defined in the USRDS (Supplementary Table 1) (10). Baseline and incident CVD were defined as at least one of the following conditions: atherosclerotic CVD (myocardial infarction, atherosclerosis, coronary artery bypass graft, percutaneous transluminal coronary angioplasty), non-atherosclerotic CVD (cardiovascular/circulatory disease, heart failure, atrial fibrillation, cerebrovascular accident/transient ischemic attack, stroke, artery disease), or other CVD (other heart disease and valve disease) in inpatient or outpatient settings. Information on hospitalization death was based on discharge diagnosis (11).

### Variables

The algorithm of one inpatient or outpatient specified diagnosis code before the index date was used to determine the presence of CKD etiology, including congenital anomalies of the kidney and urinary tract (CAKUT), non-CAKUT CKD (glomerular disease, and other CKD), and baseline conditions commonly associated with CKD as risk factors (hypertension, hyperlipidemia), co-occurrence, or consequences of the CVD conditions (Supplementary Table S1). Congenital anomalies (without CAKUT and congenital heart disease) were assessed in all the patients. Pediatric Medical Complexity Algorithm with three categories: no chronic disease, non-complex chronic disease, and complex chronic disease was employed to examine the baseline medical complexity of pediatric patients with kidney replacement therapy (12), and Charlson Comorbid Index was employed for adult patients (13).

### Statistical analysis

The time from the index date to the first occurrence of the composite outcome of CVD, censored at the date of last follow-up or the latest date in the dataset (December 31st, 2017), was described using the cumulative incidence function method with hospitalization death treated as a censored event.

Sensitivity analyses were performed to determine the timing of cardiovascular event onset. First, the rates of the composite event of CVD were calculated separately for times at risk after the index date (first receipt of dialysis) and after the index date plus 6 months for any CVD event of interest in the follow-up period. Second, stratified analyses were performed by sex, congenital anomaly, and CKD type in different age groups.

## Results

### Characteristics of the study cohort

The analytic cohort comprised 3,910 patients with dialysis aged 0–40 years, including 156 aged ≤12 years, 250 aged 13–20 years, 1,036 aged 21–30 years and 2,468 aged 31–40 years (Supplementary Figure 1). A total of 825,259 patient-dialysis receipts were analyzed for the study cohort, including 1,134 (29%) who ever had at least two modalities of dialysis and 2,776 who received single dialysis modality: 2,652 patients with hemodialysis, 26 patients with peritoneal dialysis and 98 patients received continuous renal replacement therapy.

The patient characteristics are shown in Table 1. Glomerular disease (42.92%) was the most common cause of CKD and the proportion was higher in patients aged >12 years than in younger children (14.1%). Patients with hydronephrosis and those who did not meet with a definition of CKD before the index date (37.9%) were classified into the non-CKD group. Hypertension (36.91%) and hyperlipidemia (13.94%) were commonly present at baseline, and the proportion was higher in older patients. Other common comorbid conditions, such as diabetes (27.27%) and liver disease (20.62%) were common in the 31–40 years group, whereas neurological disorders (19.87%) were prevalent in the 0–12 years group, and immunological (20.8%), gastrointestinal, and metabolic (19.6%) disorders were common in the 13–20 years group (Supplementary Table 2).

**Table 1:** Study cohort characteristics by age group.

	**Overall (*****n*** = **3,910)**		**Age group**								P-value^*^
			**0–12 years (*****n*** = **156)**		**13–20 years (*****n*** = **250)**		**21–30 years (*****n*** = **1,036)**		**31–40 years (*****n*** = **2,468)**		
**Sex**, ***n*** **(%)**											0.0016
Female	1,601	(40.95)	72	(46.15)	114	(45.60)	462	(44.59)	953	(38.61)	
Male	2,309	(59.05)	84	(53.85)	136	(54.40)	574	(55.41)	1515	(61.39)	
**CKD**, ***n*** **(%)**											< 0.0001
CAKUT	89	(2.28)	5	(3.21)	14	(5.60)	26	(2.51)	44	(1.78)	
Non-CAKUT	2,339	(59.82)	22	(14.10)	122	(48.80)	638	(61.58)	1557	(63.09)	
Glomerular disease	1,678	(42.92)	22	(14.10)	104	(41.60)	474	(45.75)	1078	(43.68)	
Non-CKD	1,482	(37.90)	129	(82.69)	114	(45.60)	372	(35.91)	867	(35.13)	
**AKI diagnosis**, ***n*** **(%)**	516	(13.20)	17	(10.90)	47	(18.80)	145	(14.00)	307	(12.44)	0.0246
**Baseline CVD**, ***n*** **(%)**	294	(7.52)	13	(8.33)	29	(11.60)	65	(6.27)	187	(7.58)	0.0375
Atherosclerotic CVD	93	(2.38)	2	(1.28)	9	(3.60)	18	(1.74)	64	(2.59)	0.1920
Non-atherosclerotic CVD	176	(4.50)	10	(6.41)	15	(6.00)	38	(3.67)	113	(4.58)	0.2273
Cardiovascular/circulatory disease	10	(0.26)	0	(0.00)	2	(0.80)	2	(0.19)	6	(0.24)	0.3236
Heart failure	95	(2.43)	1	(0.64)	4	(1.60)	24	(2.32)	66	(2.67)	0.3195
Atrial fibrillation/flutter	31	(0.79)	1	(0.64)	4	(1.60)	7	(0.68)	19	(0.77)	0.5099
Cerebrovascular/artery disease	65	(1.66)	8	(5.13)	6	(2.40)	11	(1.06)	40	(1.62)	0.0022
Other CVD	111	(2.84)	1	(0.64)	17	(6.80)	28	(2.70)	65	(2.63)	0.0006
**Hypertension**, ***n*** **(%)**	1,443	(36.91)	10	(6.41)	69	(27.60)	367	(35.42)	997	(40.40)	< .0001
**Hyperlipidemia**, ***n*** **(%)**	545	(13.94)	5	(3.21)	17	(6.80)	135	(13.03)	388	(15.72)	< .0001
In-hospital mortality event, *n* (%)	1,081	(27.65)	85	(54.49)	80	(32)	242	(23.36)	674	(27.31)	< .0001

Data are presented as counts and count (percentages). Atherosclerotic CVD: coronary artery disease (CAD) and infarction; non-atherosclerotic CVD: cardiovascular/circulatory, (congestive) heart failure, atrial fibrillation, cerebrovascular disease, artery disease; other CVD: other heart disease, valve disease.

* P-values are based on the chi-square test for categorical variables among age groups.

CKD, chronic kidney disease; CAKUT, congenital anomalies of the kidney and urinary tract; CVD, cardiovascular disease.

### Onset and cardiovascular disease risk

Overall, 7.52% of the patients had a form of specific CVD at baseline, and non-atherosclerotic CVD was more prevalent (4.5%) than other forms of CVD (Table 1). The rate of composite CVD events was 31.83% in the incident cohort (*n* = 3,616) after the initiation date of dialysis during the study period. A high rate of CVD was found in the first 6 months of follow-up (18.47%), and younger patients had a higher risk than older patients (Table 2).

**Table 2:** Incidence rate of cardiovascular disease and mortality rate by age group.

**Outcome event**	**Overall (*****n*** = **3,616)**		**Age group**								*P*-value^*^
			**0–12 years (*****n*** = **143)**		**13–20 years (*****n*** = **221)**		**21–30 years (*****n*** = **971)**		**31–40 years (*****n*** = **2,281)**		
**Within the first 6 months follow-up**											
Any CVD, *n* (%)	668	(18.47)	43	(30.07)	52	(23.53)	183	(18.85)	390	(17.1)	0.0002
Atherosclerotic CVD	146	(4.04)	6	(4.2)	8	(3.62)	39	(4.02)	93	(4.08)	0.9895
Non-atherosclerotic CVD	487	(13.47)	33	(23.08)	39	(17.65)	128	(13.18)	287	(12.58)	0.001
Cardiovascular/ circulatory disease	27	(0.75)	2	(1.4)	0		8	(0.82)	17	(0.75)	0.4644
Heart failure	255	(7.05)	10	(6.99)	15	(6.79)	72	(7.42)	158	(6.93)	0.9648
Atrial fibrillation/ flutter	99	(2.74)	8	(5.59)	6	(2.71)	27	(2.78)	58	(2.54)	0.1939
Cerebrovascular/ artery disease	158	(4.37)	17	(11.89)	24	(10.86)	33	(3.4)	84	(3.68)	< .0001
Other CVD	206	(5.70)	15	(10.49)	20	(9.05)	62	(6.39)	109	(4.78)	0.0017
**From the first 6 months to end-of-follow up**											
Any CVD, *n* (%)	483	(13.36)	12	(8.39)	24	(10.86)	126	(12.98)	321	(14.07)	0.1467
Atherosclerotic CVD	286	(7.91)	3	(2.10)	8	(3.62)	73	(7.52)	202	(8.86)	0.0016
Non-atherosclerotic CVD	339	(9.38)	12	(8.39)	16	(7.24)	90	(9.27)	221	(9.69)	0.6536
Cardiovascular/ circulatory disease	40	(1.11)	3	(2.10)	2	(0.90)	7	(0.72)	28	(1.23)	0.3929
Heart failure	188	(5.20)	5	(3.50)	15	(6.79)	51	(5.25)	117	(5.13)	0.5724
Atrial fibrillation/flutter	92	(2.54)	1	(0.70)	5	(2.26)	23	(2.37)	63	(2.76)	0.4592
Cerebrovascular/artery disease	138	(3.82)	6	(4.20)	5	(2.26)	35	(3.60)	92	(4.03)	0.5890
Other CVD	166	(4.59)	4	(2.80)	22	(9.95)	37	(3.81)	103	(4.52)	0.0007
**Over the follow-up period**											
Any CVD, *n* (%)	1,151	(31.83)	55	(38.46)	76	(34.39)	309	(31.82)	711	(31.17)	0.2591

Patients who were diagnosed with CVD before or on the date of dialysis were excluded from the incidence of composite CVD events (n = 294).

Data are presented as counts and count (percentages). Atherosclerotic CVD: coronary artery disease (CAD) and infarction; non-atherosclerotic CVD: cardiovascular/circulatory disease, (congestive) heart failure, atrial fibrillation, cerebrovascular disease, artery disease; other CVD: other heart disease and valve disease.

* P-values are based on the chi-square test for categorical variables among age groups.

**Figure 1:**
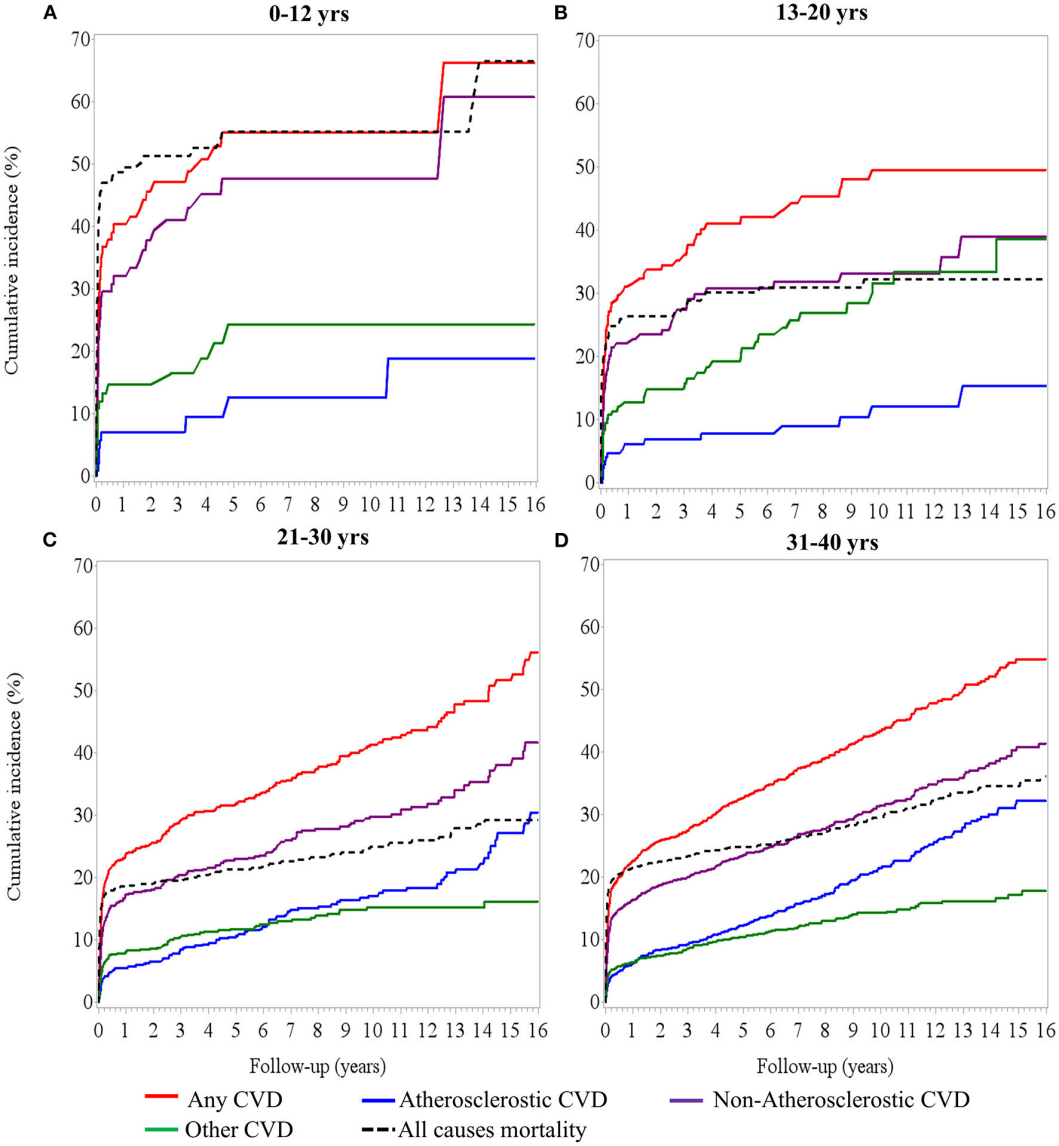
Cumulative incidence of cardiovascular event and mortality over time in **(A)** 0–12 years of age, **(B)** 13–20 years of age, **(C)** 21–30 years, and **(D)** 31–40 years of age with dialysis treatment.

Figure 1 illustrates the patterns of cumulative incidence of composite and individual types of CVD over 16 years of follow-up. The children group (0–12 years) had the highest rate of composite CVD events, especially within 6 months of dialysis (up to 40%). Non-atherosclerotic CVD, including cerebrovascular disease, heart failure, cerebrovascular disease, and arrhythmia, are the leading causes of CVD in children and remained for 16 years of follow-up (Figure 1A). The adolescent group (13–20 years) had the second highest rate of CVD (up to 30%). Despite that non-atherosclerotic CVD was still the most prevalent CVD, the incidence of other CVD, including valvular heart disease, increased progressively and became similar to the incidence of non-atherosclerotic CVD after 10 years of dialysis (Figure 1B). In young adult and adult group, non-atherosclerotic CVD remained the dominant cause of CVD; however, atherosclerotic CVD, such as coronary artery disease or myocardial infarction, increased progressively to up to 30% in these groups (Figures 1C, D).

Compared to the 31–40 years group, patients aged 0–12 years had a highest risk of developing CVD [aHR, 1.63 (1.22–2.19)], an additional 77% risk for non-atherosclerotic CVD [aHR, 1.77 (1.28–2.45)], and a 2.43-fold risk for other forms of CVD [aHR, 2.43 (1.47–4.01)] (Supplementary Figure S2). Considering the onset of CVD after 6 months of dialysis therapy in the sensitivity analysis, the rate of composite events of CVD was 16.38% (*n* = 483) in the remaining 2,948 patients at risk. The risk of composite events of CVD changed across age groups, but statistical power was insufficient to support the increased risk in those aged 0–12 years [aHR, 1.63 (0.89–2.98)] and 13–20 years [aHR, 1.47 (0.95–2.28)], compared with the 31–40 years adult group. However, young adults (21–30 years) had a significantly lower risk [aHR, 0.79 (0.64–0.98)] than the 31–40 years patient group (Supplementary Figure S2, secondary cohort analysis). These results suggest that a high rate of composited CVD events, particularly for non-atherosclerotic CVD (i.e., artery disease and other types of CVD), had faster deterioration in pediatric patients undergoing dialysis therapy in the first 6 months of follow-up.

### Risk of CVD modifiers: Sex, congenital anomaly, type of CKD

**Figure 2:**
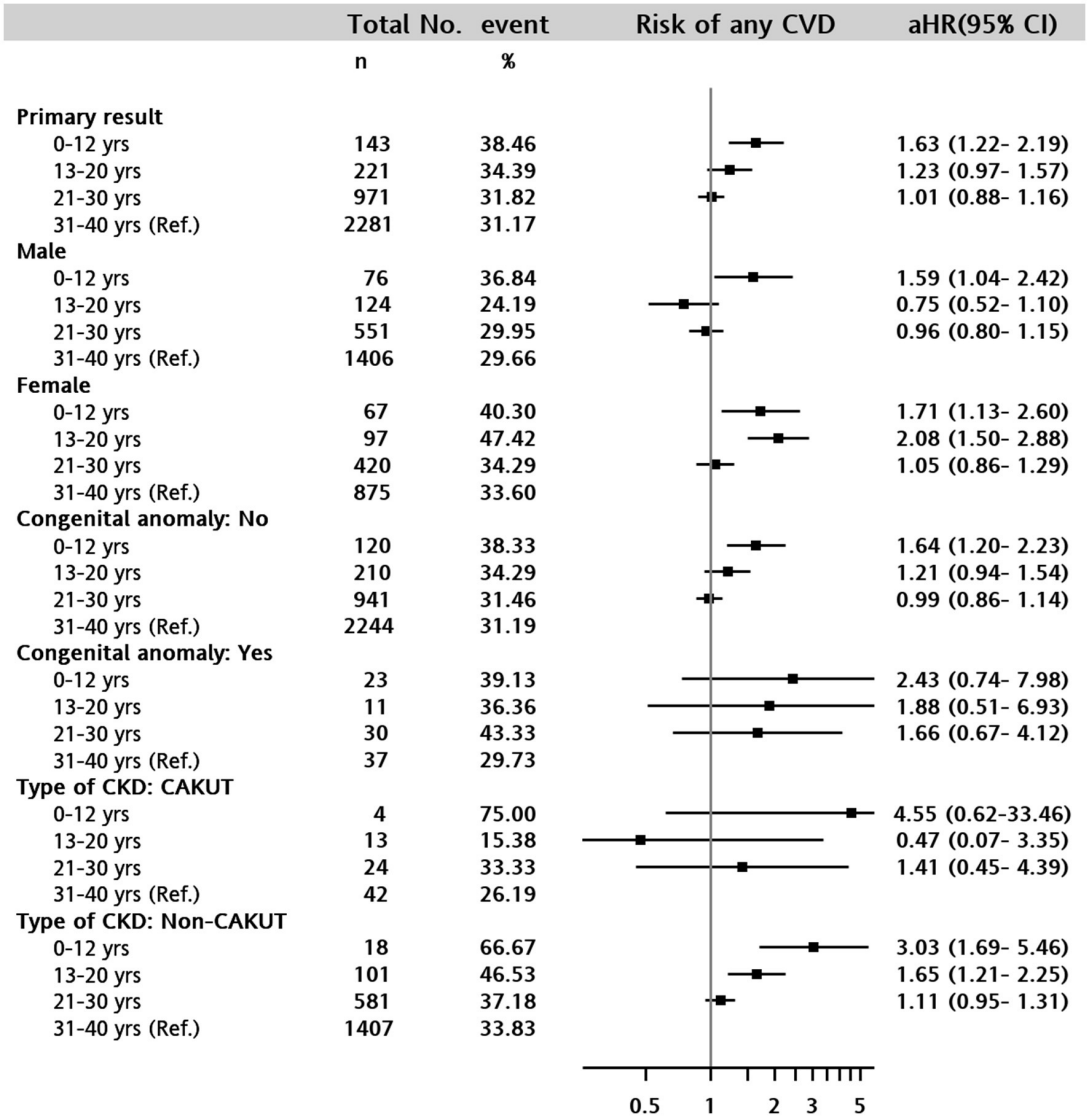
Forest plot of adjusted hazard ratio for the composite cardiovascular disease event in 0–12 years, 13–20 years, 21–30 years, and 31–40 years (reference) of age groups. The following covariates were adjusted for in the death-censored Cox proportional hazards model: Sex, type of CKD (CAKUT, non-CAKUT, and non- CKD), any congenital anomalies, baseline comorbid conditions of liver disease, severe liver disease, diabetes with and without complications, cancer, metastatic cancer, hypertension, hyperlipidemia, and AKI diagnosis.

Stratified analyses were performed to examine the individual impact of sex, congenital anomalies, and etiology of CKD on the association between ESKD and CVD. Girls showed a higher risk than boys in the age <20 years groups, particularly adolescents [aHR, 2.08 (1.5–2.88)] (Figure 2).

In the stratum without any congenital anomaly, patients in the 0–12 years group had a 1.64-fold risk of composited CVD [aHR, 1.64 (1.2–2.23)] and a 1.21-fold in the 13–20 years group [aHR, 1.21 (0.94–1.54)], compared to patients in the 31–40 years group. The statistical power was insufficient to support the impact of any congenital anomaly on age-associated CVD risks (Figure 2). In addition, non-CAKUT CKD enhanced the association between children (0–12 years) and adolescents (13–20 years) and the risk of CVD [aHR, 3.03 (1.69–5.46) and 1.65 (1.21–2.25), respectively] compared with adults in the 31–40 years group (Figure 2).

### All-cause mortality

**Figure 3:**
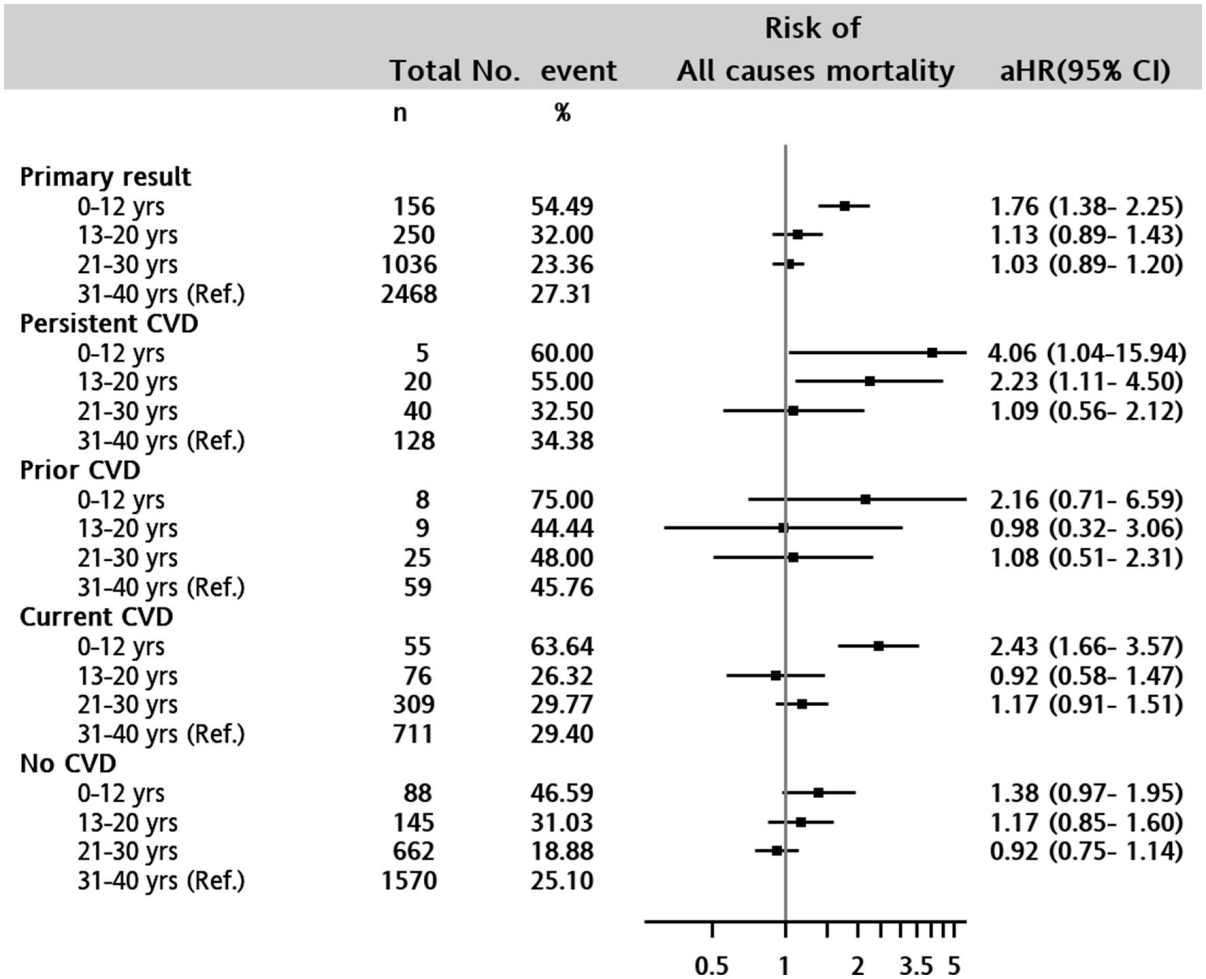
Forest plot of adjusted hazard ratio for in-hospital mortality in 0–12 years, 13–20 years, 21–30 years, and 31–40 years (reference) of age groups. Persistent CVD: patients with any CVD both baseline and during the following covariates were adjusted for in the Cox proportional hazards model: Sex, type of CKD (CAKUT, non-CAKUT, and non-CKD), any congenital anomalies, baseline comorbid conditions of liver disease, severe liver disease, diabetes with and without complications, cancer, metastatic cancer, hypertension, hyperlipidemia, AKI diagnosis, and CVD at baseline and any time before death event.

The overall in-hospital mortality rate was 27.65% (*n* = 1,081) in the entire study cohort. The association between all-cause mortality and age was significantly higher in the 0–12 years of age group [aHR, 1.76 (1.38–2.25)] and 13–20 years of age group [aHR, 1.13 (0.89–1.43)] than in the adult group (Figure 3) after controlling for baseline characteristics and presence of CVD.

**Figure 4:**
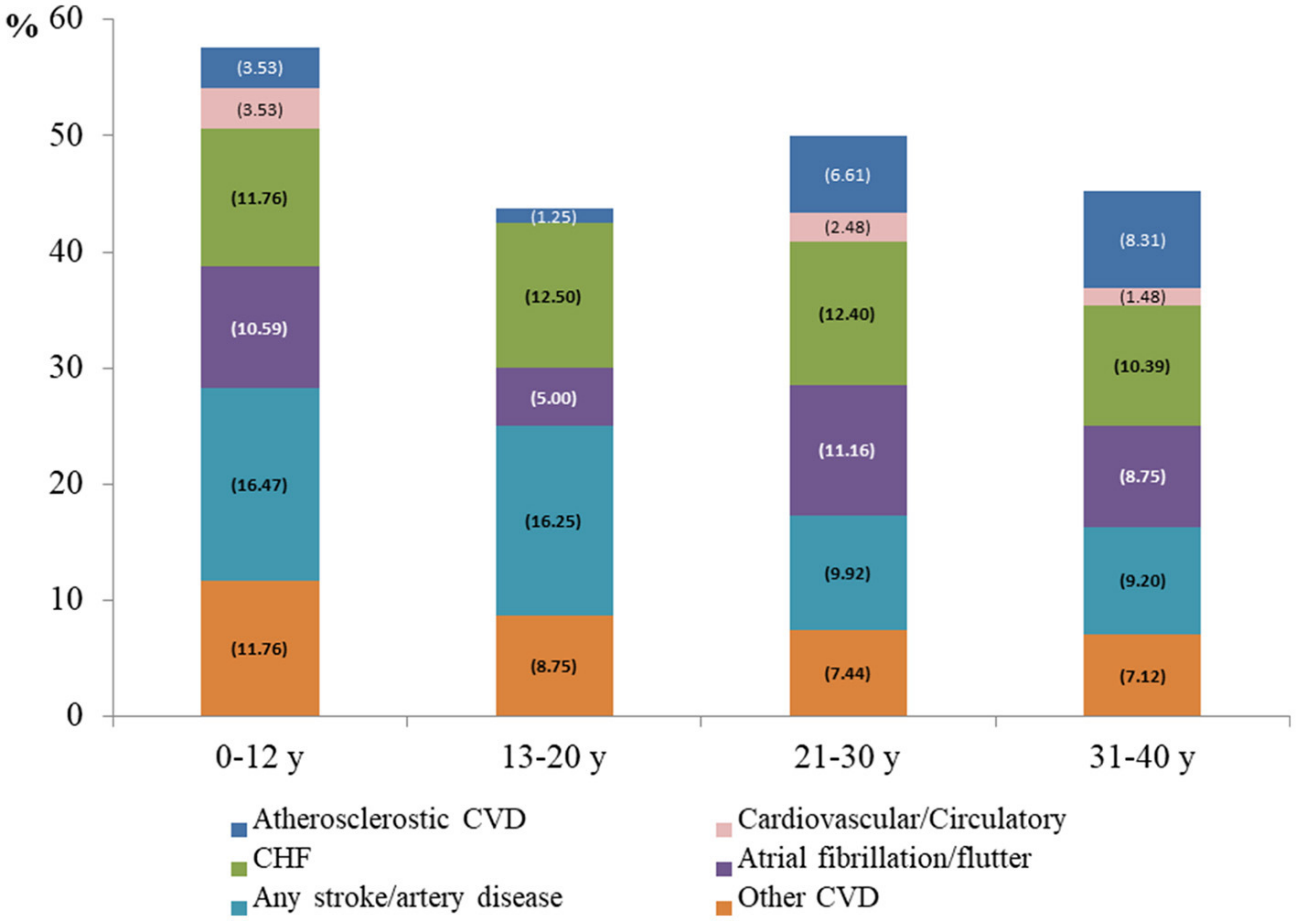
Discharge diagnosis of cardiovascular disease among patients with in-hospital mortality Atherosclerotic CVD, coronary artery disease and infarction; Non-atherosclerotic CVD, cardiovascular/circulatory, (congestive) heart failure, atrial fibrillation/flutter, any stroke, artery disease; other CVD, other heart disease and valve disease.

Of the 1,081 patients with a hospital death event, 34.69% (*n* = 375) had a CVD diagnosis at discharge (7.03% with atherosclerotic and 28.31% with non-atherosclerotic CVD). The rate of any CVD diagnosis was higher in children aged <12 years (*n* = 39, 45.88%), followed by 13–20 years (*n* = 27, 33.75%), 21–30 years (*n* = 94, 38.84%) and 31–40 years (*n* = 215, 31.9%). Visually, the all-cause mortality risk was close to that of non-atherosclerotic CVD in the 13–20 years and 21–30 years age groups over time (Figure 1). Figure 4 shows that (congestive) heart failure was the most common diagnosis, accompanied by in-hospital mortality, and was independently associated with age. However, arterial disease and other CVD were more prevalent in children and adolescents than in adults, details of CVD at discharge are listed in Supplementary Table 3. Other common disease conditions were liver failure and related diseases, acute kidney injury, sepsis, pneumonia, kidney failure, gastrointestinal bleeding, fluid overload, and electrolyte imbalance, which were prevalent at hospital discharge among patients with in-hospital death events.

## Discussion

In the current study, we compared the morbidity of CVD onset in four age groups when dialysis therapy was initiated. Dialysis treatment initiated in pediatric patients carries a greater risk of mortality and CVD than that in adults. In general, non-atherosclerotic CVD was more prevalent, especially in younger patients, within the first 6 months after the initiation of dialysis. After 6 months of initial dialysis, the risk of atherosclerotic CVD was higher in adults than that in adolescents and children. Girls showed a higher risk of CVD than boys in the 13–20 years age group.

Previous reports have shown that CVD-related mortality can be up to 500 times and even one thousand times higher than in age-matched young adults (10) and children (14), respectively. Our results supported that pediatric patients had a higher mortality rate than older patients. Furthermore, it is critical to delineate the etiologies of CVD in different age groups at the time of dialysis initiation. In the current study, children had the highest rate of all-cause mortality and composite CVD events especially at the initiation (within 6 month) of dialysis. Non-atherosclerotic CVD, such as heart failure or arrhythmia (atrial fibrillation/flutter), are the leading causes of CVD and the most prevalent diagnosis accompanying death events in children. After 6 months, the incidence of CVD has become less common in children than that in adolescents and adults, which is consistent with a previous publication from USRDS (10). In another study from USRDS 2006–2008, the causes of cardiac death in children 0–19 years of age with CKD were cardiac arrest/arrhythmia, followed by cerebrovascular diseases and heart failure (15). Unlike older adults, children generally have neither diabetes nor symptomatic atherosclerosis at the time of CKD diagnosis. Previous studies have shown that left ventricular (LV) hypertrophy and LV dysfunction occur early in CKD, and heart failure and atrial fibrillation may appear early after dialysis initiation (16, 17). CV abnormalities develop early and progress during the course of CKD, eventually leading to high mortality and CV events at the beginning of ESKD in children.

In addition to CVD, underlying diseases may also contribute to the high mortality in children. Of note, all-cause mortality rate was higher than composite CVD in the first 3 years and then became close to all CVD afterwards (Figure 1A). In contrast, a recent study using data from the USRDS (10) demonstrated a lower CVD incidence in children than in young adults. This discrepancy may be explained because our data originated from medical centers, where patients tended to have more comorbidities, such as liver diseases and cancer, which may also be responsible for the high mortality observed in the children. Moreover, although CAKUT CKD is prevalent in childhood ESKD (18), the role of CAKUT in CVD remains unclear. Our study showed that CAKUT played a minor role compared to other CKDs in the progression of CVD. Other non-CAKUT related CKDs contribute more to CVD development in children.

In the group aged 13–20 years, the incidence of CVD was approximately 23.5% at the beginning of dialysis therapy, which is lower than that in children but higher than that in adults. Non-atherosclerotic CVD is the most prevalent CVD. However, the incidence of other CVD, including other heart and valve diseases, increased progressively and catch up non-atherosclerotic CVD after 10 years of dialysis. Notably, girls showed a higher risk of CVD than boys, particularly in this age group. The etiology of CKD in this group is more glomerular disease (19), associated with the highest connective tissue disease, immunological, neurological, and pulmonary diseases. Autoimmune or connective tissue disease-related glomerulonephropathy, such as IgA nephropathy (8) or systemic lupus erythematosus (20), contributes to CKD in adolescents, especially in girls. Inflammation plays an important role in autoimmune and systemic-related CKD and CVD. The treatment of underlying diseases and screening for underlying CVD may help control mortality in adolescents.

In young adults and adults undergoing dialysis, non-atherosclerotic CVD remains the dominant cause of CVD. However, atherosclerotic CVD, such as coronary artery disease or myocardial infarction, increased progressively to up to 30% in these groups. The most common diseases associated with atherosclerotic CVD are diabetes, hypertension, and hyperlipidemia, which represent the traditional risks of CVD in the general population (21). Moreover, other CKD-related factors such as uremic toxins, vascular calcification, protein-energy waste, systemic inflammation, and oxidative stress are also important risk factors for CVD, and the influence of these factors may outweigh the traditional factors in advanced CKD or even post-dialysis (21–24). Unlike children, the incidence rates of CVD and mortality in the group aged 20–40 years were the lowest (<20%) at the beginning of ESKD but increased progressively to 50% and 30%, respectively, after more than 10 years of dialysis (Figures 1C, D). The control of traditional and non-traditional CKD-related risk factors for atherosclerotic CVD may help reduce the long-term CVD burden in young adults and adults.

The strength of the current study is the accurate quantification of the prevalence and incidence of cardiovascular events for different age groups at the time of dialysis therapy, to compare cardiovascular risk in a large dialysis cohort aged <40 years, and to explore atherosclerotic and non-atherosclerotic CVD-specific risk factors. Our findings have important implications for the assessment of cardiovascular risk in CKD patients. First, high CV events and mortality rates occur in the early stages of dialysis in children, and non-atherosclerotic CV events, such as heart failure, arrhythmia, and cerebrovascular diseases, are the main contributors to mortality. Second, as dialysis therapy proceeds, the role of atherosclerosis in CVD becomes increasingly important in young and older adults. Both traditional and CKD-related non-traditional risk factors for atherosclerotic CVD contribute to the etiology of CVD in these groups.

This study had certain limitations. First, CVD ascertainment relies on routinely collected electronic health record data in a healthcare delivery system, which may underestimate the prevalence and incidence of CVD. Second, based on the demographics of the study cohort and national health insurance program-supported healthcare system in Taiwan, our findings may not be generalizable to other countries.

## Conclusion

The rates of cardiovascular morbidity and mortality were high and varied in different age groups, which remind clinicians to apply different strategies for CVD and mortality prevention in patients of different ages requiring chronic dialysis.

## Data availability statement

The original contributions presented in the study are included in the article/Supplementary material, further inquiries can be directed to the corresponding author.

## Ethics statement

The studies involving human participants were reviewed and approved by the Institutional Review Board of the Chang Gung Medical Foundation in Taipei, Taiwan. Written informed consent from the participants' legal guardian/next of kin was not required to participate in this study in accordance with the national legislation and the institutional requirements.

## Author contributions

Research idea, study design, data analysis, and interpretation: L-CL, C-NH, and Y-LT. Data acquisition: C-NH. Statistical analysis: H-CK. Supervision or mentorship: C-NH and Y-LT. Each author contributed important intellectual content during manuscript drafting or revision and agrees to be personally accountable for the individual's own contributions and to ensure that questions pertaining to the accuracy or integrity of any portion of the work, even one in which the author was not directly involved, are appropriately investigated and resolved, including with documentation in the literature if appropriate. All authors contributed to the article and approved the submitted version.
