# Supplementary material for: Cardiovascular diseases morbidity and mortality among children, adolescents and young adults with dialysis therapy
Source: Front Public Health. 2023 Apr 12;11:1142414. doi: 10.3389/fpubh.2023.1142414 (PMC10130397; doi:10.3389/fpubh.2023.1142414)
Supplement: Supplementary file 1 [file Table_1.DOCX]

Supplementary Material

Cardiovascular diseases morbidity and mortality among children, adolescents and young adults with dialysis therapy

SUPPLEMENTARY TABLE 1. Codes and algorithms for disease conditions and procedures in the dialysis cohort

SUPPLEMENTARY TABLE 2. Baseline comorbid conditions in the study cohort

SUPPLEMENTARY TABLE 3. Patterns of cardiovascular disease at discharge in patients with all-cause mortality events

SUPPLEMENTARY FIGURE 1. Patient selection process

SUPPLEMENTARY FIGURE 2. Forest plot of adjusted hazard ratio for the composite cardiovascular disease event after the first 6 months post dialysis therapy initiation in four age groups. When considering the CVD events occurring within the first 6 months as existing health status, the secondary cohort (n=2948) demonstrated a similar ranking of CVD hazard across the age groups.

**SUPPLEMENTARY TABLE1. Codes and algorithms for disease condition and procedure in the dialysis cohort**

| Health condition | Code | |
| --- | --- | --- |
| Dialysis therapy: ≥ 2 receipts in a year | | |
| Hemodialysis | Billing code: 58001C，58019C，58020C， 58021C，58022C，58023C，58024C，58025C，58029C | |
| Peritoneal dialysis | Billing code: 58002C，58009A，58009B，58010A，58010B，58011A，58011AB，58011B，58011C， 58012A，58012B，58017B，58017C，58026C，58028C | |
| Cardiovascular disease (CVD): ≥ 1 codes for the diagnosis in inpatient or outpatient setting | | |
| **Atherosclerotic CVD (coronary artery disease)** | | |
| Myocardial infarction | ICD9:410, 411,412,413, 414 | ICD10:I20, I21, I22, I23, I24, I25 |
| Atherosclerosis | ICD9:440, 441,442,443,444,445, 447, 448 | ICD10:I70-I79 |
| Coronary artery bypass graft | Billing code:68023.X (wild code), 68024.X, 68025.X, 68053B, 68054B, 68055B | |
| Percutaneous transluminal coronary angioplasty | Billing code: M22-047, M22-311, M22-312, M22-313, M22-314, M22-315 | |
| **Non-Atherosclerotic CVD** | | |
| Cardiovascular/circulatory disease | ICD9:429.2,429.79, 459 | ICD10:I519, I513, I230,I233, I236,I237,I238, I87, I99 |
| (Congestive) Heart failure | ICD9:398，422，425，428，402， 404 | ICD10:I09.81，I40-I43，I50，I11，I13 |
| Cerebrovascular disease/transient ischemic attack | ICD9:430–438 | ICD10:I60-I63，I65-I69，G45-G46。 |
| Artery disease | ICD9:442, 443, 444.21, 446,447, | ICD10:I72, I73, I742, I77,I79, M30, M31 |
| **Other CVD** | | |
| Other heart disease | ICD9:420,422,423,425,429, 416 | ICD10:I23, I27, I30,I31, I32, I40, I41, I42, I43, I51, I52, I971 |
| Congenital heart disease | ICD9:745, 746, 747, | ICD10:Q20-Q28 |
| Valve disease | ICD9:394, 395, 396, 397, 391, 424, 424.9,421, V433, | ICD10:I01, I05-I09, I33-I39, Z952 |
| **Comorbid conditions** | | |
| Hypertension | ICD9:401-405 | ICD10:I10~I13,I15, N26.2 |
| Hyperlipidemia | ICD9:272 | ICD10:E75.2, E75.3, E75.5, E75.6, E77, E78, E88.1, E88.2, E88.89 |
| **Chronic kidney disease (CKD)** : ≥ 1 codes for the diagnosis in inpatient or ≥ 2 codes in outpatient setting; patients with any CAKUT and non-CAKUT were classified as CAKUT; | | |
| Congenital anomalies of the kidney and urinary tract(CAKUT) | ICD9: 589, 593.71, 593.72, 596.0 (excl. 753.6), 753, 753.0, 753.1, 753.2, 753.20, 753.21, 753.22, 753.29, 753.3, 753.4, 753.6, 753.8, 753.9, | ICD10: N270, N271, N279, N13721, N13731, N13722, N13732, N320, Q60, Q61, Q62,Q63, Q64 |
| Glomerular disease | ICD9: 580, 581, 582, 583, 28311 | ICD10: N00, N01, N02, N03, N04, N05, N06, N07, N08, D593 |
| Other CKD* | ICD9: 250.4, 255.1, 272.7, 274.1, 403.1, 442.1, 572.4, 585, 586, 587, 588, 588.1 588.8, 588.9, 96.54, 710.0, 791.0 | ICD10: E1021, E1022, E1029, E1065, E1121, E1122, E1129, E1165, E260, E268, E261, E269, E752, E753, E770, E771, E778, E779, M1030, M10311, M10312, M10319, E1032, M1033, M1034, M1035, M1036, E1037, M1038, M1039, N13, N200, M1030, I129, I120, I722, I7773, K767, N140, N141, N142, N143, N144, N150, N158, N159, N184, N185. N186, N189, N19, N261, N269, N250, N251, N2581, N2589, N259, N310, N311, N319, M320, M3210, M3219, M328, M329, R800, R801, R803, R808, R809 |

* Hydronephrosis was included as CKD in the USRDS, which was classified as non-CKD in this study. Reference: Modi ZJ, Lu Y, Ji N, et al. Risk of cardiovascular disease and mortality in young adults with end-stage renal disease: an analysis of the US Renal Data System. 2019;4(4):353-362.

**SUPPLEMENTARY TABLE2. Baseline comorbid conditions for the study cohort**

| Baseline comorbidity | | | Overall  (n=3910) | | Age group | | | | | | | | | | | *p-value** |
| --- | --- | --- | --- | --- | --- | --- | --- | --- | --- | --- | --- | --- | --- | --- | --- | --- |
|  |  |  |  |  | 0-12 years (n=156) | |  | 13-20 years (n=250) | |  | 21-30 years (n=1,036) | |  | 31-40 years (n=2,468) | |  |
| **Any congenital anomalies, n (%)** | | | 113 | (2.89) | 27 | (17.31) |  | 12 | (4.80) |  | 32 | (3.09) |  | 42 | (1.70) | <.0001 |
|  | Ear/Nose/Throat | | 12 | | 4 | (2.56) |  | 3 | (1.20) |  | 1 | (0.10) |  | 4 | (0.16) | <.0001 |
|  | Respiratory | | 9 | | 6 | (3.85) |  | 1 | (0.40) |  | 2 | (0.19) |  | 0 | (0.00) | <.0001 |
|  | Digestive/Gastrointestinal | | 8 | | 4 | (2.56) |  | 1 | (0.40) |  | 2 | (0.19) |  | 1 | (0.04) | <.0001 |
|  | Musculoskeletal | | 11 | | 6 | (3.85) |  | 2 | (0.80) |  | 1 | (0.10) |  | 2 | (0.08) | <.0001 |
|  | Skin/Dermatology | | 14 | | 0 | (0.00) |  | 1 | (0.40) |  | 5 | (0.48) |  | 8 | (0.32) | 0.7763 |
|  | Chromosomes | | 8 | | 1 | (0.64) |  | 1 | (0.40) |  | 3 | (0.29) |  | 3 | (0.12) | 0.3731 |
|  | Others/Unspecific | | 61 | | 9 | (5.77) |  | 5 | (2.00) |  | 22 | (2.12) |  | 25 | (1.01) | <.0001 |
| **Carlson comorbid index, n (%)** | | | | |  |  |  |  |  |  |  |  |  |  |  |  |
|  |  | Acute myocardial infarction |  | |  |  |  |  |  |  | 2 | (0.19) |  | 30 | (1.22) |  |
|  |  | Congestive heart failure |  | |  |  |  |  |  |  | 75 | (7.24) |  | 230 | (9.32) |  |
|  |  | Peripheral vascular diseases |  | |  |  |  |  |  |  | 13 | (1.25) |  | 56 | (2.27) |  |
|  |  | Cerebral vascular accident |  | |  |  |  |  |  |  | 36 | (3.47) |  | 114 | (4.62) |  |
|  |  | Dementia |  | |  |  |  |  |  |  | 2 | (0.19) |  | 9 | (0.36) |  |
|  |  | Pulmonary disease |  | |  |  |  |  |  |  | 60 | (5.79) |  | 155 | (6.28) |  |
|  |  | Connective tissue disorder |  | |  |  |  |  |  |  | 126 | (12.16) |  | 158 | (6.40) |  |
|  |  | Peptic ulcer |  | |  |  |  |  |  |  | 94 | (9.07) |  | 335 | (13.57) |  |
|  |  | Liver diseases |  | |  |  |  |  |  |  | 97 | (9.36) |  | 509 | (20.62) |  |
|  |  | Diabetes mellitus (DM) |  | |  |  |  |  |  |  | 100 | (9.65) |  | 427 | (17.30) |  |
|  |  | DM complications |  | |  |  |  |  |  |  | 61 | (5.89) |  | 246 | (9.97) |  |
|  |  | Paraplegia |  | |  |  |  |  |  |  | 12 | (1.16) |  | 22 | (0.89) |  |
|  |  | Renal disease |  | |  |  |  |  |  |  | 611 | (58.98) |  | 1567 | (63.49) |  |
|  |  | Cancer |  | |  |  |  |  |  |  | 40 | (3.86) |  | 197 | (7.98) |  |
|  |  | Severe liver diseases |  | |  |  |  |  |  |  | 11 | (1.06) |  | 105 | (4.25) |  |
|  |  | Metastatic cancer |  | |  |  |  |  |  |  | 6 | (0.58) |  | 53 | (2.15) |  |
|  | **Pediatric medical complexity algorithm (PMCA), n (%)** | | | | | | | | | | | | | | | |
|  | Non-Chronic | | 796 | | 91 | (58.33) |  | 69 | (27.60) |  |  |  |  |  |  |  |
|  | Non-complex Chronic | | 133 | | 9 | (5.77) |  | 12 | (4.80) |  |  |  |  |  |  |  |
|  | Complex Chronic | | 2981 | | 56 | (35.90) |  | 169 | (67.60) |  |  |  |  |  |  |  |
|  |  | Cardiac | 1615 | | 15 | (9.62) |  | 74 | (29.60) |  |  |  |  |  |  |  |
|  |  | Craniofacial | 5 | | 1 | (0.64) |  | 1 | (0.40) |  |  |  |  |  |  |  |
|  |  | Dermatological | 61 | | 2 | (1.28) |  | 0 | (0.00) |  |  |  |  |  |  |  |
|  |  | Endocrinological | 880 | | 5 | (3.21) |  | 20 | (8.00) |  |  |  |  |  |  |  |
|  |  | Gastrointestinal | 920 | | 17 | (10.90) |  | 49 | (19.60) |  |  |  |  |  |  |  |
|  |  | Genetic | 32 | | 4 | (2.56) |  | 4 | (1.60) |  |  |  |  |  |  |  |
|  |  | Genitourinary | 198 | | 5 | (3.21) |  | 18 | (7.20) |  |  |  |  |  |  |  |
|  |  | Hematological | 492 | | 23 | (14.74) |  | 44 | (17.60) |  |  |  |  |  |  |  |
|  |  | Immunological | 466 | | 6 | (3.85) |  | 52 | (20.80) |  |  |  |  |  |  |  |
|  |  | Malignancy | 281 | | 9 | (5.77) |  | 24 | (9.60) |  |  |  |  |  |  |  |
|  |  | Mental health | 260 | | 13 | (8.33) |  | 20 | (8.00) |  |  |  |  |  |  |  |
|  |  | Metabolic | 855 | | 21 | (13.46) |  | 49 | (19.60) |  |  |  |  |  |  |  |
|  |  | Musculoskeletal | 214 | | 10 | (6.41) |  | 12 | (4.80) |  |  |  |  |  |  |  |
|  |  | Neurological | 553 | | 31 | (19.87) |  | 40 | (16.00) |  |  |  |  |  |  |  |
|  |  | Ophthalmological | 432 | | 8 | (5.13) |  | 19 | (7.60) |  |  |  |  |  |  |  |
|  |  | Otologic | 181 | | 2 | (1.28) |  | 5 | (2.00) |  |  |  |  |  |  |  |
|  |  | Pulmonary/Respiratory | 220 | | 15 | (9.62) |  | 37 | (14.80) |  |  |  |  |  |  |  |
|  |  | Renal disease | 2375 | | 26 | (16.67) |  | 124 | (49.60) |  |  |  |  |  |  |  |
|  |  | Progressive | 2835 | | 46 | (29.49) |  | 156 | (62.40) |  |  |  |  |  |  |  |
|  | Diabetes mellitus | |  | |  |  |  |  |  |  |  |  |  |  |  |  |
|  |  | Type I | 139 | | 1 | (0.64) |  | 2 | (0.80) |  |  |  |  |  |  |  |
|  |  | Type II | 521 | | 1 | (0.64) |  | 5 | (2.00) |  |  |  |  |  |  |  |

Data are presented as counts and percentages.

**P* values were based on Chi-square test for categorical variables among age groups

References:

Rattanasompattikul M, Feroze U, Molnar MZ, et al. Charlson comorbidity score is a strong predictor of mortality in hemodialysis patients. *Int Urol Nephrol.* 2012;44(6):1813-1823.

Simon TD, Cawthon ML, Stanford S, et al. Pediatric medical complexity algorithm: a new method to stratify children by medical complexity. *Pediatrics.* 2014;133(6):e1647-1654.

**SUPPLEMENTARY TABLE 3.** **Patterns of cardiovascular disease at discharge in patients with all-cause mortality events**

|  | | Overall  (n=1081) | |  | Age group | | | | | | | | | | | *P value** |
| --- | --- | --- | --- | --- | --- | --- | --- | --- | --- | --- | --- | --- | --- | --- | --- | --- |
|  |  |  |  |  | 0-12 years (n=85) | |  | 13-20 years (n=32) | |  | 21-30 years (n=242) | |  | 31-40 years (n=674) | |  |
| Any CVD diagnosis, n(%) | | 375 | (34.69) |  | 39 | (45.88) |  | 27 | (33.75) |  | 94 | (38.84) |  | 215 | (31.90) | 0.0308 |
| Atherosclerotic CVD | | 76 | (7.03) |  | 3 | (3.53) |  | 1 | (1.25) |  | 16 | (6.61) |  | 56 | (8.31) | 0.0593 |
| Non-Atherosclerotic CVD | | 306 | (28.31) |  | 33 | (38.82) |  | 23 | (28.75) |  | 75 | (30.99) |  | 175 | (25.96) | 0.0623 |
|  | Cardiovascular/circulatory | 19 | (1.76) |  | 3 | (3.53) |  | 0 | (0.00) |  | 6 | (2.48) |  | 10 | (1.48) | 0.2615 |
|  | Heart failure | 120 | (11.10) |  | 10 | (11.76) |  | 10 | (12.50) |  | 30 | (12.40) |  | 70 | (10.39) | 0.8115 |
|  | Atrial fibrillation/flutter | 99 | (9.16) |  | 9 | (10.59) |  | 4 | (5.00) |  | 27 | (11.16) |  | 59 | (8.75) | 0.3667 |
|  | Any stroke/artery disease | 113 | (10.45) |  | 14 | (16.47) |  | 13 | (16.25) |  | 24 | (9.92) |  | 62 | (9.20) | 0.0611 |
| Other CVD (heart disease/valve disease) | | 83 | (7.68) |  | 10 | (11.76) |  | 7 | (8.75) |  | 18 | (7.44) |  | 48 | (7.12) | 0.4851 |

Atherosclerotic CVD: coronary artery disease (CAD) and infarction; Non-atherosclerotic CVD: cardiovascular/circulatory, heart failure, atrial fibrillation, cerebrovascular disease (any stroke), artery disease; Other CVD: other heart disease, valve disease, congenital heart disease

**P* values were based on Chi-square test for categorical variables among age groups; data are same as Figure 4


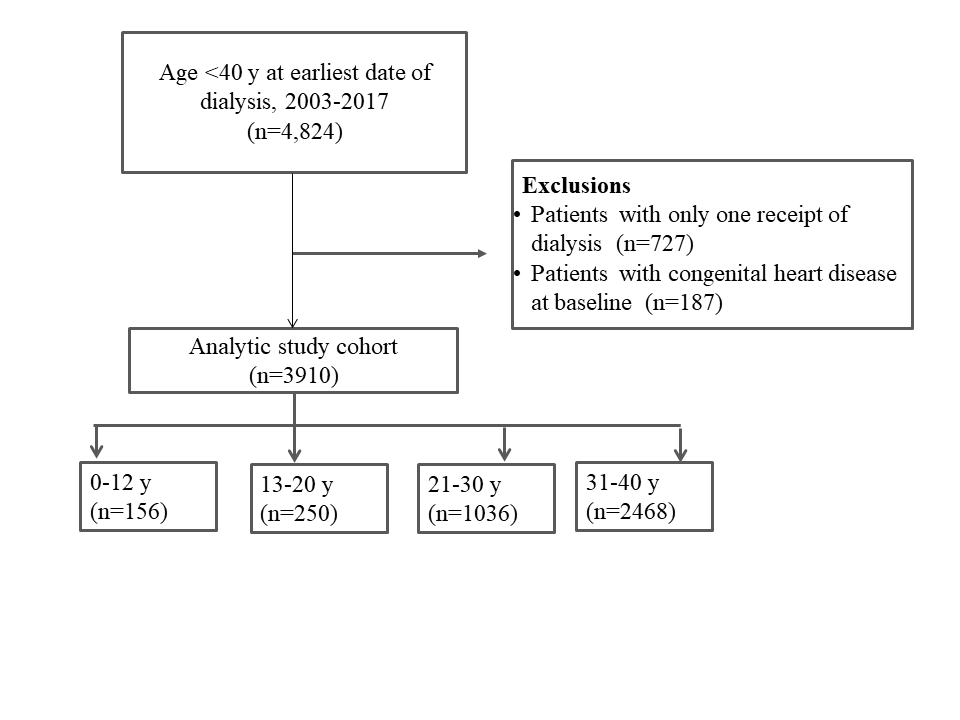


**SUPPLEMENTARY FIGURE 1. Patient selection flowchart**

| 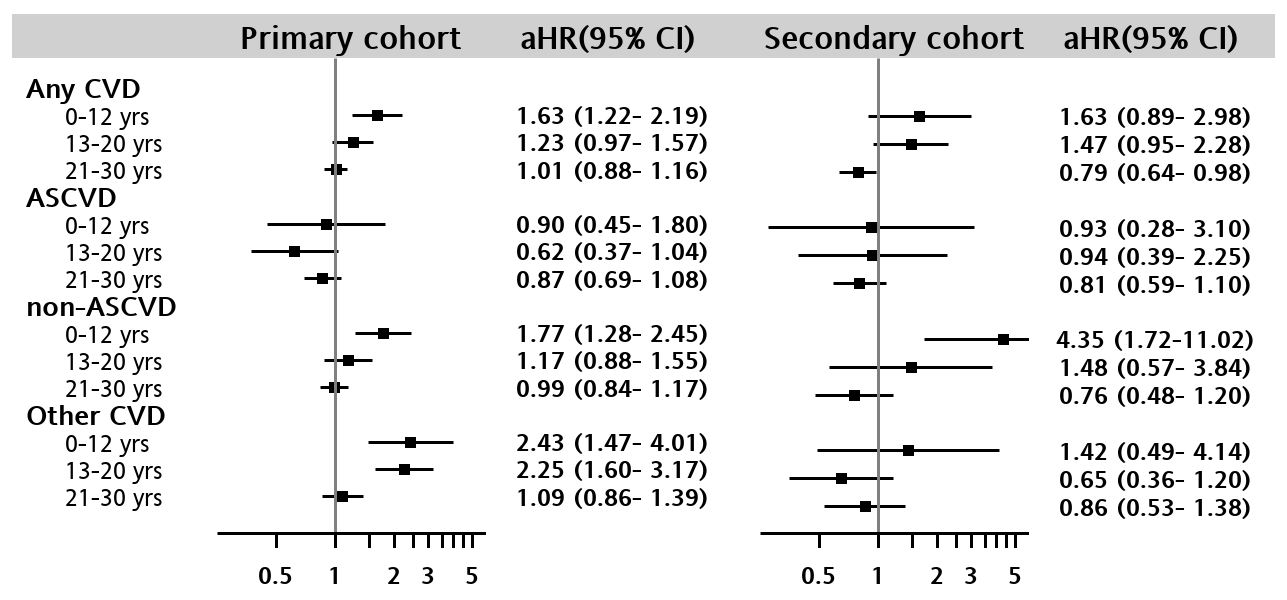 |
| --- |
| **SUPPLEMENTARY FIGURE 2.** Forest plot of adjusted hazard ratio for the composite cardiovascular disease event after the first 6 months post dialysis therapy initiation in four age groups When considering the CVD event occurred within the first 6 months as existed health status, the secondary cohort (n=2948) demonstrated a similar ranking of hazard of CVD across age groups |
